# What Variations in the Manner of Using the Teeth Are Exhibited by the Various Animals Including Man

**Published:** 1889-05

**Authors:** F. S. Buckley

**Affiliations:** W. of M.


### What Variations in the Manner of Using the Teeth Are Exhibited by the Various Animals Including Man.

BY F. S. BUCKLEY,

W. OF M.

The present paper includes the teeth of batrachians and reptiles and three mammalian orders, edentates, cetaceans, and ungulates. Among the batrachians the frog has only one row of simple pointed teeth upon the upper jaw; the lower jaw is edentulous. These are merely prehensile teeth and not masticatory. The toad is edentulous, seizing the beetles, caterpillars, slugs, etc., forming its food, between its jaws and sometimes uses its fore paws to push the struggling insects down its throat. The frog and toad simply swallow the animals used for food and in the case of the toad they usually reach the stomach uninjured.

Among the reptiles the tortoises and turtles have no teeth, but are provided with horny cases upon the margins of the jaws, sharp-edged in carnivorous, and blunt-edged in herbivorous species.

The lizards have conical teeth, sharp and pointed in some, and blunt and rounded in other species; these teeth are, however, always used in seizing the insects used as food. One lizard, the New Zealand Hatteria, uses the margins of the jaws as a masticating surface after the teeth are worn down. This is one of very few instances where bone, uncovered by any dental tissue, is used for mastication.

Snakes may be divided into venomous and non-venomous. The non-venomous snakes have two rows of teeth upon the upper jaw and one row upon the lower jaw, sharp, curved teeth, pointing inwards and backwards, and firmly anchylosed to the jaws. These are used precisely as the teeth of the fishes, for *seizing* the prey, and in no sense for masticating purposes, the animal being swallowed whole and alive, of whatever size or shape. The boa constrictor and anaconda, however, squeeze larger animals to death before swallowing, which is accomplished by advancing first the lower jaw, then the upper, and thus forcing itself over the body of its prey. Among the venomous serpents some have poison fangs constantly erect and only slightly longer than the other teeth, but the viper, adder, rattlesnake and others have very highly specialized poison fangs. These are very long and curved teeth, and lie flat along the roof of the mouth when not in use and are only erected when the snake strikes. When this occurs the contraction of the muscles of the face causes the huge poison glands to be compressed and the secretion to flow out at the base of the poison fang from which it is directed into a canal in the tooth by a fold of mucus membrane surrounding its base, and discharged through the opening near the apex of the tooth while plunged into the flesh of its victim.

Crocodiles and alligators have large, sharp, conical teeth used only for seizing and tearing their prey, which is not swallowed whole as in serpents and fishes, but often consists of very large masses of flesh which are torn into pieces of convenient size for swallowing.

Snakes and lizards have sharp conical teeth when in the egg shell, developed only for the purpose of cutting through the shell, and are lost soon after the exit of the animal. Crocodiles use the hard snout for this purpose. Among the extinct reptiles are many very peculiar varieties of teeth whose especial use is only imperfectly known.

Existing birds have no teeth but use horny bills or beaks for the same purposes. H›wever, if it were possible here to consider the teeth of the several very peculiar species of extinct birds as they may be studied from fossil remains, we should find several species with true teeth. These are reptilian in character and indicate clearly the carnivorous nature of the food used, as well as the fact that these birds use their teeth ouly as prehensile organs, and in no true sense masticate their food.

The mammalian teeth are of the highest structure and have the most varied uses. Excepting the edentates, mammals have four kinds of teeth in the same mouth, each having a special function to perform; whereas we have seen that all animals lower than the mammals have but one kind of teeth.

The edentates have simple conical teeth for seizing and tearing all kinds of food, animal and vegetable, excepting the South American ant eater, which, in the absence of teeth, uses its long tongue, coated with a viscid secretion, as a prehensile organ.

Among the cetaceans the dolphin has about two hundred slender, sharp, conical teeth for seizing its prey. The sperm and bottle-nosed whales have small, regular, almost rudimentary teeth; the whalebone whale has no teeth at all, but entangles the small marine animals forming its food in the fringes of the whalebone plates, and then sweeping them into the throat with the tongue—swallows them. The narwal has a very peculiar tusk from ten to twelve feet long in the upper jaw on the left side. The right tusk is buried in the jaw bone; as also in the female. These are the only teeth of the narwal and the long tusk is probably a sexual weapon.

The ungulates, or hoofed animals are all herbivorous and have teeth conforming closely to the mammalian type and modified according to the different vegetable forms upon which they subsist. The grinding surfaces of the premolars and molars have complex patterns formed by the enamel dipping deeply into the crown and preserving constantly a roughened surface for trituration, because of the unequal density and resistance to wear of the enamel and dentine. The cuspids and incisors are often used as weapons and as prehensile organs.

The existing odd-toed ungulates, comprising the rhinoceros, tapir, and horse have four premolars and three molars of large size and complex grinding surface, presenting probably the most perfect triturating surface among living animals. The cuspids are stunted because of their slight use and are absent entirely in the rhinoceros. The incisors are prehensile organs for biting off grass and herbs. Those of the horse are very large and strong, meeting end to end, and therefore wearing down rapidly. Instead of having enlarged cuspids in the shape of tusks, like the wild boar, the horse uses its very strong incisors as weapons.

The existing even-toed ungulates, comprising the hippopotamus, camel, sheep, ox, antelope, and deer, have molars used about the same as in the horse, etc. The incisors vary greatly in use.

The wild boar has large cuspid tusks used as sexual weapons. In the male Sus barbirussa the upper canine tusks grow to great length, pierce the upper lip and curve backward toward the eyes. Their use is not clearly understood, although evidently a sexual organ, but are not much used in fighting, and they have been known to grow directly into the head of the animal.

The hippopotamus uses its incisor and canine tusks for uprooting aquatic plants which it eats, and also as weapons of offense.

The sheep, ox, and antelope have no canine teeth using their horns as offensive and defensive weapons, but there are three species of deer which have large canines, apparently because of the smallness of their horns. In fact, it seems to be a rule to find canines developed among the deer when sufficient protection is not afforded by the horns. The incisors are absent in the upper jaw, the animal being unable to bite, but tears off the blades of grass by a peculiar movement of the head. The existing ungulates are only a small proportion of those once inhabiting the earth which possessed very interesting and peculiar teeth, whose uses are, however, very little known.

Article II.—The orders of the animal kingdom which have not been considered in the preceding part of this paper are the sirenia, proboscidia, rodentia, carnivora, insectivora, chiroptera, and primates, which latter includes man.

The only two animals of the order sirenia are the dugong and manatee which present one great variation in the use of their teeth, in that they do not use them at all. The dugong has eight or ten, the manatee twelve rudimentary incisors and cuspids buried in the lower jaw and covered in with horny plates. These are distorted and stunted teeth which are never erupted and are eventually absorbed. However, both animals have good molars for masticating the seaweed and aquatic plants upon which they live.

The proboscidians, comprising the elephant, mastodon, and dinotherium, of which, however, only the elephant is now living. Its dentition is peculiar to itself. The two incisor tusks projecting from the upper jaw are the only teeth anterior to its molars, of which it has only one in use at any time on each side of each jaw. These tusks are usually sexual teeth, although the female of the African elephant has as large tusks as the male. They form the weapon of the elephant in defense and attack; they are also used in uprooting grass which is roughly shaken before being taken into the mouth to remove the adherent earth. The molars, of the ungulate type, are kept uneven by the constant trituration of grass and roots thus mingled with earthy matter.

The elephant also bites off the limbs of juicy succulent trees and eats them without using its tusks at all. Tame elephants use the tusks for many purposes, as for instance, in pulling a rope the rope is passed over the tusk and then seized between the molar teeth to give a purchase.

The rodents present variations, especially in the use of the incisor teeth, of which they have only four, two in the upper and two in the lower jaw, very long, strong persistently growing teeth. The articulating surfaces of the lower jaw are very long antero-posteriorly and permit a very great backward and forward motion as used in gnawing. The molar teeth vary according to the nature of the food used, whether animal, vegetable, or a combination of both. The beaver illustrates the uses of the incisors of the rodents. They will gnaw through a tree eighteen inches in diameter and then drag such portions of it as they cut off to their dams, and filling it with brush and earth, form a secure dam to running streams. They also dig and gnaw away earth and roots, forming a canal to transport their timbers to the refuge pond where they live.

In the carnivora the cuspid teeth are the ones especially developed and the incisors uniformly small. The molars are also multi-cuspid and more like many cuspid teeth than molars. The result of this conformation is that the animal food is seized, torn and swallowed in chunks and masses of flesh. No mastication is really effected and a dog will swallow the mass of meat given him as rapidly as he can tear it into pieces small enough to pass the throat. This is the general use of the teeth of the carnivora, while in all of them the enlarged cuspid teeth are the principal weapons of offense.

Some, however, eat vegetable food as well as animal and hence we see in the bear true masticating surfaces on the molars to masticate the berries which it consumes as part of its food. Among the seals the walrus presents the greatest peculiarity in its upper canines, which are very long and pass down far below the lower jaw. These are used both in the male and female in clambering over ice and tearing up marine plants and turning over obstacles which prevent their seeing the crustaceans upon the sea bottom. They are also the weapons of the walrus.

Insectivora have multi-cuspid molars and, in general, teeth adapted to seizing and tearing the small insects forming their food.

Among the chiroptera the vampire has only one incisor on each side of the upper jaw with which the wound is made and its molars are stunted as we should expect in an animal living only on blood.

The teeth of the monkey are much like those of man, comprising all the kinds of teeth necessary for mastication where a mixed diet is found. There is no especial variation in the teeth or manner of using; but the cuspid teeth are enlarged in the male, almost forming a sexual weapon.

The teeth of man are the most perfect to be found in the animal kingdom and have the most varied uses. In the matter of eating the teeth of man are adapted for both animal and vegetable food and are evidently intended for very thorough work in masticating hard foods and meats. The manner of the articulation of the upper and lower bicuspids and molars enables these teeth to reduce the solid food to very small particles prior to its entrance into the stomach. As a weapon the teeth are not used except in a state of deepest barbarism and where brutal passion overrules every suggestion of reason and judgment; in short, the teeth and nails are only used when man becomes a mere animal. The teeth of man are not used as prehensile organs to any extent, the hands and fingers conveying the food to the mouth. Man is the only animal which uses the teeth for other than their appropriate uses.

While the uses of the teeth of all other animals are clearly defined and adhered to with the simplicity of the animal instinct, the reason of man, perverted and warped to suit his abnormal desires, indicates the most peculiar and absurd uses for the teeth, so that we find men using the teeth in a variety of ways, which are sometimes simply amusing, sometimes very injurious.

The teeth of all animals doubtless discharge the function of giving their share of expression to the face, but this use of the teeth is hardly thought of in connection with any animal but man for the reason that no other animal as frequently loses its teeth and thus destroys the normal expression of the lower part of the face completely. This is often the case with man's teeth and indicates very clearly the immense importance of the teeth in giving expression to the face.

Probably the highest use of the teeth of man is that of assisting in the articulation of sounds, in words, as the expression of thought and emotion. Man uses the teeth, mouth, throat, and vocal organs in this process of speaking, and this is the distinguishing characteristic which separates man from all other members of the animal kingdom.

---

IT WASN'T HIS TOOTH.—Countryman to Dentist.—I wouldn't pay nothing extra for gas. Just lug her out. Never mind if it does hurt.

Dentist.—Well, you are plucky, sir. Let me see the tooth.

Countryman.—Oh, 'tain't me that's got the toothache; it's my wife. She will be here in a minute.

